# Plasma cupping induces VEGF expression in skin cells through nitric oxide-mediated activation of hypoxia inducible factor 1

**DOI:** 10.1038/s41598-019-40086-8

**Published:** 2019-03-07

**Authors:** Hyun-Young Lee, Hae-June Lee, Gyoo-Cheon Kim, Jeong-Hae Choi, Jin-Woo Hong

**Affiliations:** 1 Department of electrical engineering, Pusan National University, Busan, South Korea; 2 Department of Anatomy and Cell Biology, School of Dentistry, Pusan National University, Yangsan, South Korea; 3 Department of Internal Medicine, School of Korean Medicine, Pusan National University, Yangsan, South Korea

## Abstract

Despite a long history, the clinical efficacy of cupping therapy is still under debate. This is likely due to the lack of direct evidence for the biological actions of cupping, since the short exposure of cells to vacuum condition rarely has affects cellular activity. In this study, the medicinal properties of a recent medical technology, non-thermal plasma, were added to classical cupping and designated as ‘plasma cupping’ (PC). In our results, the plasma-generating efficacy was increased under a cupping-like semi-vacuum condition (410 Torr) rather than normal atmospheric pressure (760 Torr). Notably, while cupping rarely affects the angiogenic factor vascular-endothelial growth factor (VEGF)-A, the PC treatment on HaCaT human keratinocytes significantly induced the expression of VEGF-A. The increased expression of the VEGF-A gene after the PC treatment was expected to be a result of PC-mediated ERK protein activation. The PC-mediated activation of ERK was essential for the activity of hypoxia inducible factor (HIF) 1 alpha, which is responsible for the PC-mediated expression of VEGF-A. The PC mediated increase of NO in the media was thought as a main reason for the elevated HIF-1 protein activity. In addition to the angiogenesis-promoting action of PC, it also showed anti-inflammatory activity by reducing TNF-α-mediated IL-1β and IL-6 expression. Taken together, this study indicates the potential for PC that could enhance the clinical efficacy of cupping by adding the effects of non-thermal plasma to traditional cupping.

## Introduction

Cupping therapy is one of the oldest alternative medical procedures, along with acupuncture, with more than 3500 years of history^1,2^ to treat pain and various disorders. There are several types of cupping therapy, but it can be divided into two styles: wet cupping and dry cupping^3^. Dry cupping simply uses vacuum force on the surface of the skin. Conversely, wet cupping involves creating tiny wounds on the skin before the cupping procedure, and so the therapy is accompanied by the loss of blood from the wound. In both types of cupping, most of the medicinal effects might be due to several biological changes after the formation of a vacuum on the skin surface within the cup. Traditionally, the vacuum within the cup on the skin surface was formed by heat^4^. Before the cup would be placed on the skin surface, the air inside the cup is warmed by flaming or boiling the cup. After placing the heated cup, the suction force formed naturally as the air temperature of the cup decreased. Currently, the electronic cupping device does not use heat for creating a vacuum within the cup, and the cup material can be substituted with plastic wear instead of glass. Dry cupping is frequently used to relieve several kinds of pain including muscle pain^1^. Despite a long history, the effectiveness of cupping therapy is still under debate. This skepticism for cupping might come from a lack of scientific evidence for direct medicinal effects of cupping. Recently, Lowe presented a possible role for the dry cupping technique in their detailed review article^5^. In line with other believers of cupping, he insisted that various biological responses could be evoked by suction of the skin. About 5 to 10 minutes of cupping causes extravascular blood within the subcutaneous tissue and creates bruise-like marks. Since this phenomenon was caused by vacuum-mediated rupture of capillaries, it differs from trauma-mediated bruising that accompanies vascular tissue damage. Subsequently, by inducing a process to remove exposed blood components, cupping may help to repair the injured subcutaneous tissue by identifying them as if they were injured. Therefore, cupping induces mild damage at a painful part of the body and accelerates healing by evoking the natural healing process. While this hypothesis is acceptable for some people, it might be not enough to change the minds of skeptics since cupping itself has no curative effects. If the healing process after cupping can be accelerated by merging cupping with scientifically proven techniques, this new form of therapy might help to persuade skeptics on the efficacy of cupping.

Meanwhile, non-thermal plasma (NTP) has been introduced as a new form of medicinal technique. In physics, the term ‘plasma’ represents the ionized gas state. When excessive energy or heat was added to neutral state gases, the electrons of the matter can depart, and the gas can be ionized. During this process, many working elements such as UVs, electrons, ions, and reactive species (reactive oxygen of nitrogen species) can be generated. Therefore, the use of plasma is regarded as an ‘all-in-one’ treatment of these elements. This feature of plasma had been initially adapted to metal working and semiconductor production using high-temperature plasma. About two decades ago, the NTP generating technique had been developed, and several biological and medicinal effects of NTP began to be elucidated^6^. Currently, the strong anti-bacterial effects of NTP along with promotion of wound healing have been widely studied^7–9^. NTP can also enhance the effectiveness of transdermal drug delivery^10^, and recent reports support the possibility that NTP might be helpful to treating various type of cancers^11^. Furthermore, in our previous reports, the strong anti-inflammatory effect of NTP was suggested under an atopic dermatitis mice model^12^. Therefore, if these medicinal effects of NTP can be merged with cupping, this new technique of plasma cupping (PC) might not only strengthen the clinical benefits of traditional cupping but also will expand the applicable range of cupping therapy.

In our previous study, the treatment of skin cells with NTP generated by using argon gas as the vehicle gas effectively induced the angiogenesis-related gene, vascular endothelial growth factor A (VEGF-A)^13^. Since this function of NTP might help the effectiveness of cupping, the effects of cupping and NTP generated under normal atmospheric pressure (760 Torr) or cupping conditions (410 Torr) on VEGF-A expression in HaCaT human keratinocytes were tested and compared. In addition, to analyze the specific mechanism for plasma cupping on VEGF-A regulation, western blot assays were performed. To test the anti-inflammatory activity of PC, the effect of PC on tumor necrosis factor alpha (TNF-α)-mediated interleukin 1β and 6 was examined. Taken together, this study identifies PC as a new type of cupping combined with several medicinal functions of NTP.

## Results

### Plasma Cupping (PC) significantly induces the expression of VEGF-A gene

**Figure 1:**
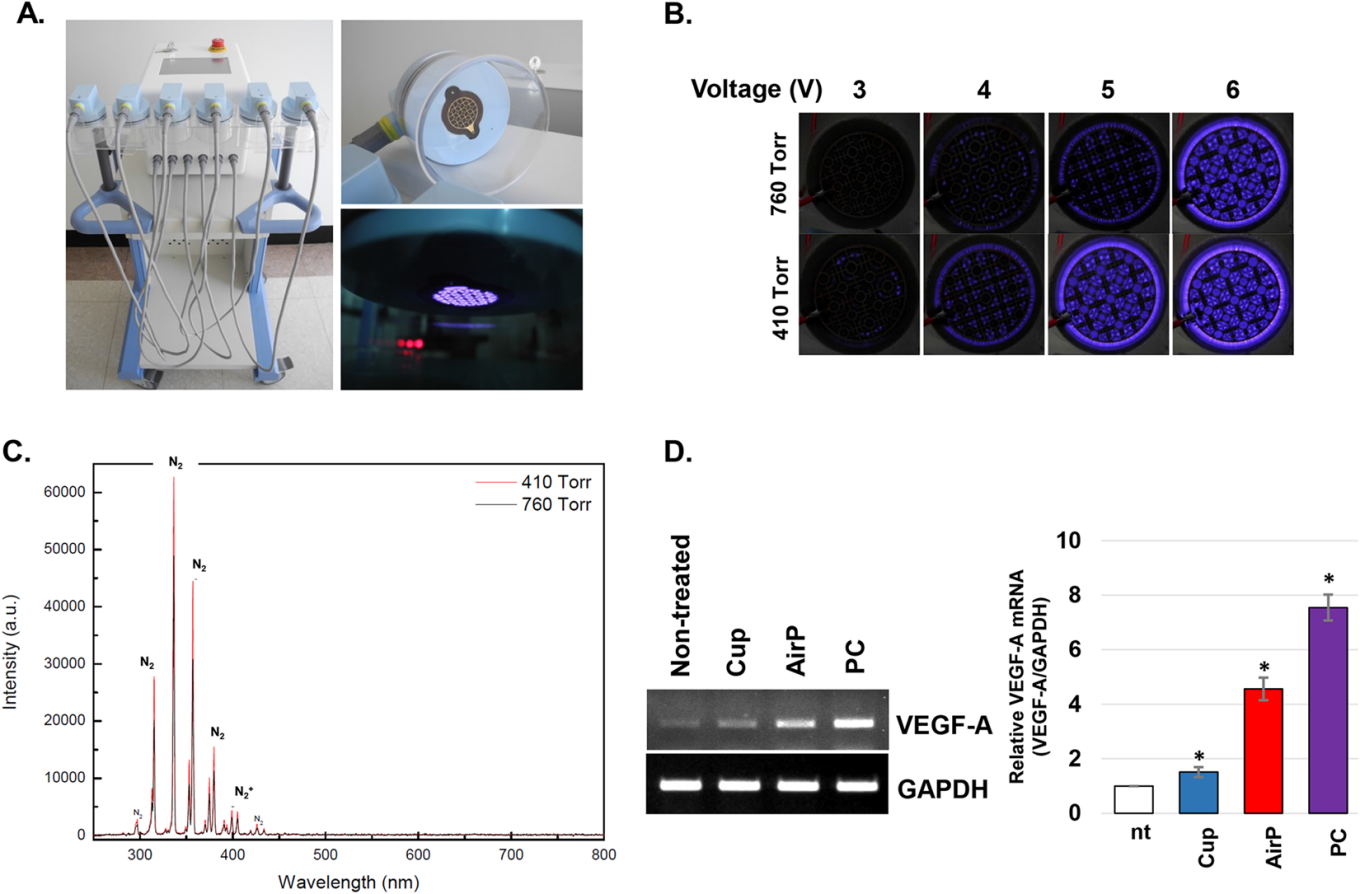
Plasma-generating efficiency can be increased under the cupping condition, and promote VEGF-A gene expression. (**A**) The photographs of the PC device developed and used in this study. **(B)** The photographs showing the plasma-generating efficiency under the normal (760 Torr) and cupping (410 Torr) condition when 3, 4, 5, and 6 V were applied as input power. **(C)** The result of OES analysis of the plasma generated under the normal (760 Torr, black peaks) and cupping (410 Torr, red peaks) condition. **(D)** The results of RT-PCR analysis. HaCaT human keratinocytes were treated with cupping (Cup), air plasma (airP), and plasma cupping (PC) for 5 minutes. After 6 hours of further incubation, total RNA from the cells were used for the RT-PCR assay. Data shown are representatives of three independent experiments, *p < 0.05.

To test plasma generation efficiency under the pressure associated with cupping (410 Torr) and normal atmospheric pressure (760 Torr), the light emitting intensity from the plasma-generating module was photographed. When the input voltage was 3 V (output voltage: 5.2 kV_pp_), plasma generation from the module was not detected under the normal condition, but the plasma ejecting dimming light was generated from the module under the cupping condition (Fig. 1B). When 4 V of input voltage was applied (output voltage: 6.78 kV_pp_), plasma started to be generated under the normal condition, but the light intensity from the module was brighter under the cupping condition. This different efficacy of plasma generation under normal and cupping conditions was the most clear when the input power was 5 V (output voltage: 8.2 kV_pp_), while the gap was reduced when 6 V (output voltage: 9.6 kV_pp_) was applied. Based on these results, the most effective PC condition, 5 V of input power and 410 Torr, was determined and used for the following experiments in this study.

In order to analyze the plasma intensity and chemical composition of plasma under the normal condition (airP) and PC, the OES analysis was utilized. As our data shows, the airP did not directly produce reactive oxygen species (ROS) or reactive nitrogen species (RNS) (Fig. 1C). However, the peaks of reactive N_2_, the secondary N_2_ system, along with N_2_^+^ were generated effectively. The OES data for PC also indicated that it did not directly produce ROS or RNS from the module, but the level of excited N_2_ was elevated compared to that of the airP.

To compare the effects of cupping, airP, and PC on the expression of VEGF-A, HaCaT human keratinocytes were subjected to cupping, airP, and PC for 5 minutes. After 6 hours of incubation, the expression of VEGF-A gene was analyzed. As Fig. 1D shows, the treatment of cells with cupping slightly induced VEGF-A gene expression (about 1.6 fold) compared to un-treated cells. Conversely, the treatment of cells with airP resulted in a 4.3-fold increase in VEGF-A gene expression, while PC treatment elevated the expression by about 7.8 fold.

### PC treatment on HaCaT cells slightly induced HIF-1α protein expression along with activation of ERK and JNK kinases

**Figure 2:**
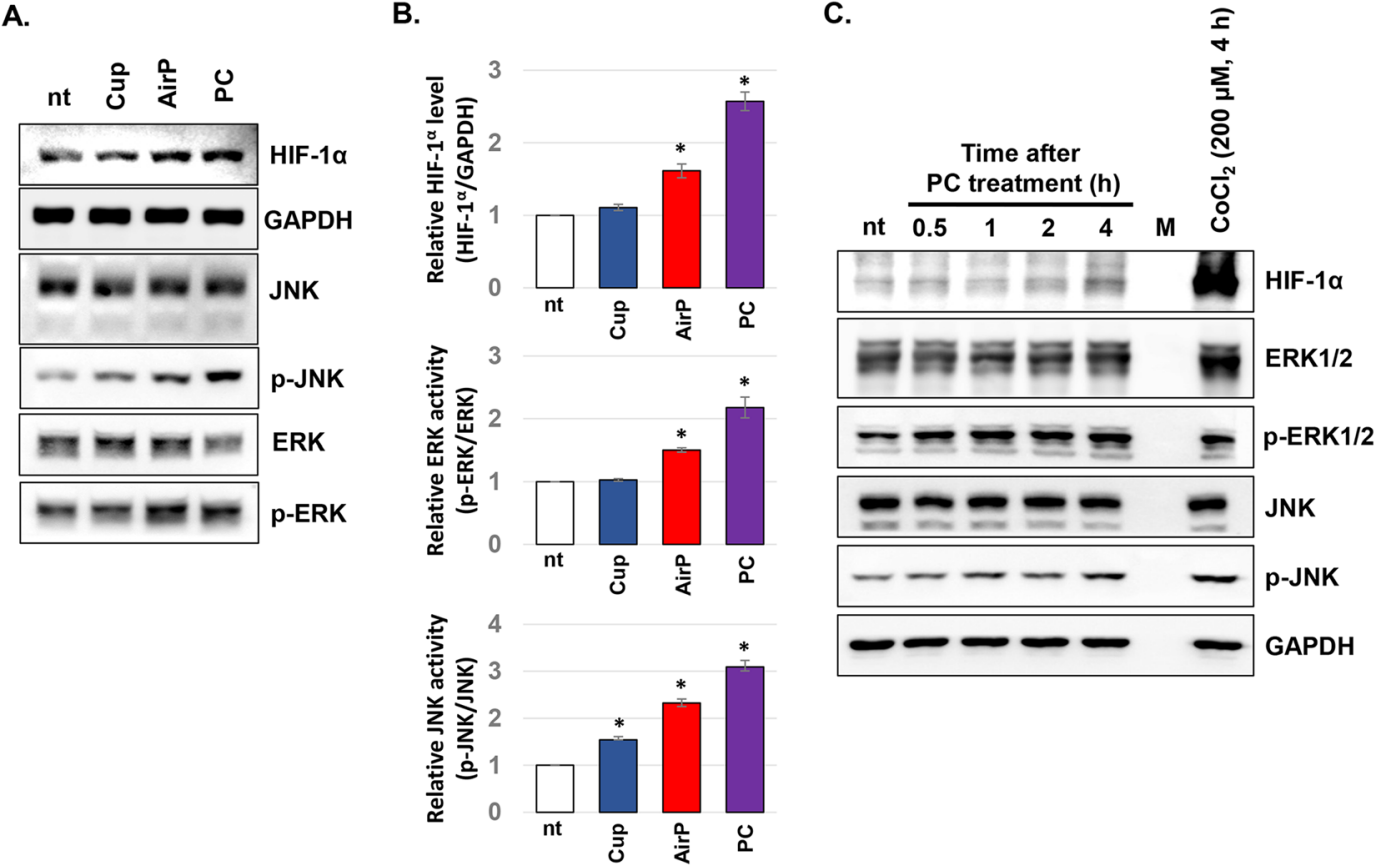
PC increased the protein level of HIF-1α along with the activity of ERK and JNK. (**A,B**) HaCaT cells were subjected to Cup, AirP, and PC treatment for 5 minutes. After 4 hours of incubation, total cell lysates were used for western blot analysis. The representative figures of three independent experiments were presented **(A)**, and the protein levels of HIF-1α as well as the activity of ERK and JNK proteins were calculated using Image J program, averaged, and presented as graphs **(B)**. Data shown are representatives of three independent experiments, * p < 0.05. **(C)** At 0.5, 1, 2, and 4 hours before the harvest, HaCaT cells were treated with PC for 5 minutes. Cobalt chloride (CoCl_2_) was used as a control for HIF-1α protein stabilization. After indicated incubation times, the total cell lysates of the samples were used for western blot assay. Data shown are the representatives of three independent experiments.

The effects of cupping, airP, and PC on protein expression were tested further to elucidate the mechanism for VEGF-A induction. For this, HaCaT cells were subjected to cupping, airP, and PC treatment for 5 minutes. After 4 hours of further incubation, the expression levels of HIF-1α, ERK, and JNK along with their protein activity was analyzed by performing western blot analysis (Fig. 2A,B). Interestingly, the treatment of cells with mere cupping did not significantly affect the HIF-1α protein level, but airP and PC increased the levels by about 1.7 fold and 2.6 fold, respectively. Furthermore, airP and PC also induced the activity of ERK and JNK proteins. Specifically, the cupping treatment failed to activate ERK protein, but airP and PC significantly induced ERK activity (about 1.5 and 2.1 fold, respectively). Meanwhile, the activity of JNK was stimulated in all treatments with cupping, airP, and PC by about 1.5, 2.2, and 3 fold, respectively.

To monitor the precise effect of PC on ERK, JNK, and HIF-1α, the cells were treated with PC for 5 minutes at 0.5, 1, 2, and 4 hours before the cell harvest. Then, total cell lysates were subjected to western blot analysis. In order to compare the effect of PC with a well-known HIF-1α protein inducer, the cells were also treated with 200 μM of CoCl_2_ for 4 hours and used as positive control. As Fig. 2C shows, the PC-mediated induction of HIF-1α protein was detected at 4 hours after the PC treatment, but this induction was relatively smaller compared to the effects of CoCl_2_. Meanwhile, the PC-mediated ERK activation was detected from 30 minutes after the PC treatment, and its effectiveness at 4 hours after the treatment was stronger than that of CoCl_2_. Conversely, an increase in JNK protein activity was detected from 1 hour after the PC treatment, but it was weaker than CoCl_2_-mediated activation.

### PC increases the expression of VEGF-A gene though ROS-mediated activation of ERK

**Figure 3:**
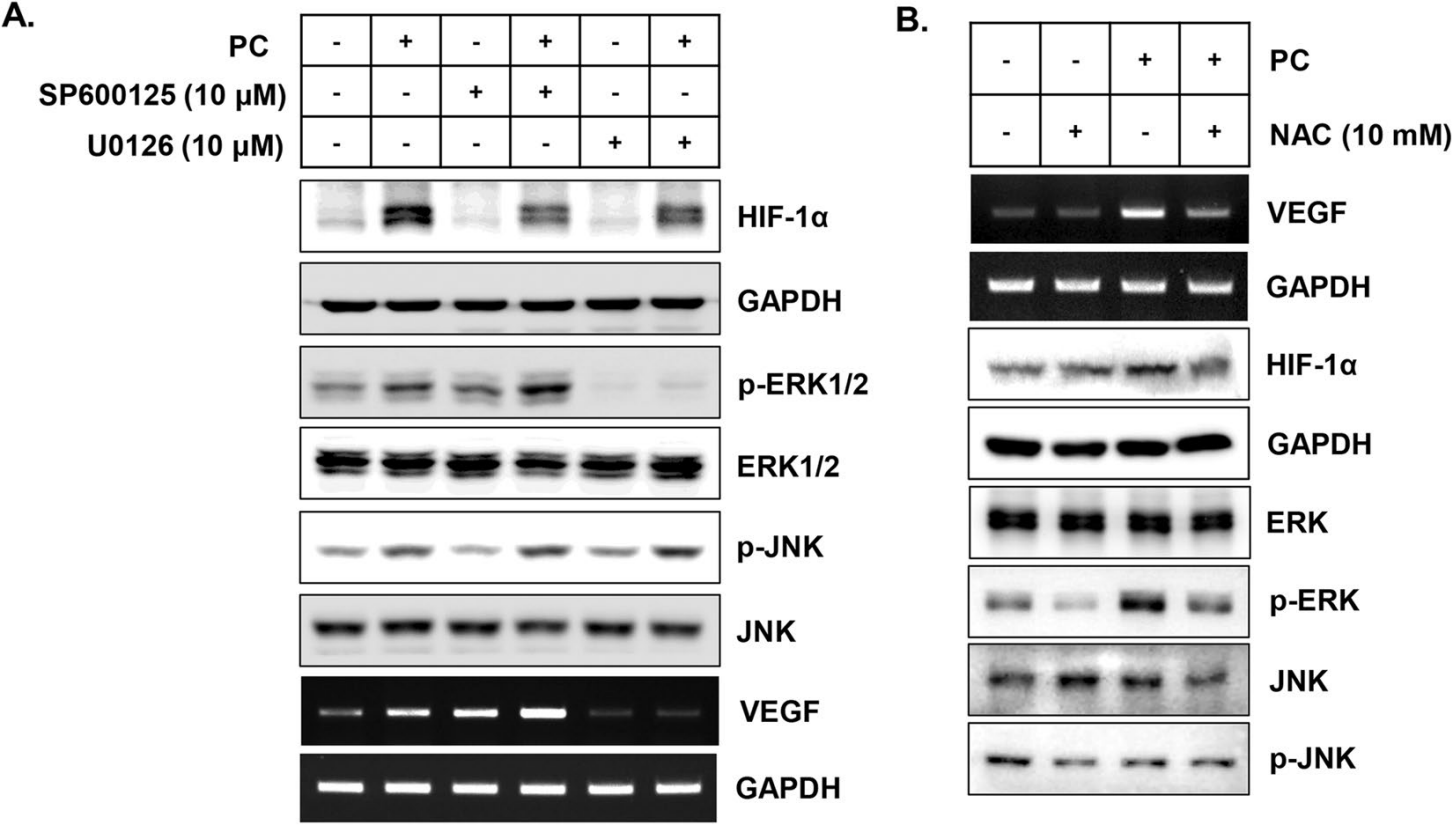
The ROS-dependent ERK activation is critical for the PC-mediated induction of VEGF-A gene expression. (**A**) HaCaT cells were pre-treated with media containing 10 μM of specific inhibitors for either JNK (SP600125) or ERK (U0126) for 30 minutes, and then the cells were subjected to PC treatment for 5 minutes. After 4 hours (for western blot, top 6 panels) or 6 hours (for RT-PCR, bottom 2 panels) of further incubation, cells were harvested, and total protein or RNA from the cells was used for the following experiments. **(B)** Before the PC treatment, HaCaT cells were incubated at 10 mM of NAC-containing growth media for 30 minutes. After 4 or 6 hours of incubation, total protein or RNA from the cells were used for western blot or RT-PCR assay, respectively. Data shown are the representatives of three independent experiments.

To elucidate the role of PC-mediated activation of two kinases, ERK and JNK, on the expression of VEGF-A gene and HIF-1α protein, the responses of HaCaT cells against PC treatment were monitored in the presence and absence of either the specific inhibitor of ERK, U0126, or the inhibitor of JNK, SP600125. As Fig. 3A shows, the PC treatment under the JNK-inactivated condition slightly reduced PC-mediated induction of HIF-1α, but the expression of VEGF-A was increased. Along with VEGF-A, the activity of ERK1 was elevated by SP600125; interestingly, it was further increased by PC treatment. Conversely, use of the ERK inhibitor completely blocked the PC-mediated induction of VEGF-A, but its effect on HIF-1α was not severe.

Several studies elucidated that the plasma-mediated increase in intracellular ROS is one of the key elements for several biological functions of the plasma. To test the role of ROS on the PC-mediated increase in VEGF-A, HaCaT cells were treated with the pan-ROS scavenger NAC, and the cellular response after PC treatment was monitored (Fig. 3B). Our data showed that NAC treatment successfully blocked the PC-mediated increase in VEGF-A gene and HIF-1α protein expression, along with PC-mediated ERK activation.

### PC-mediated activation of HIF-1α is essential for the increase in VEGF-A gene expression

**Figure 4:**
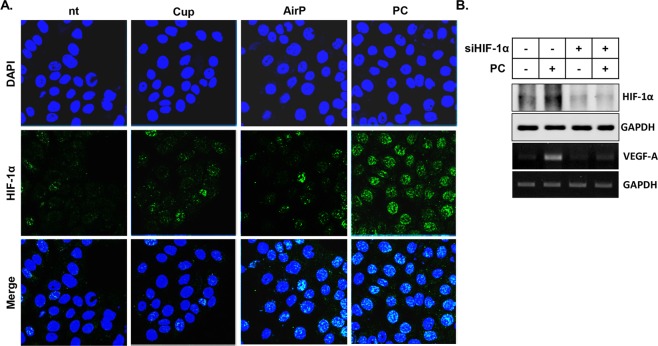
HIF-1 protein activity is critical for the PC-mediated VEGF-A gene expression. **(A)** The results of IF against HIF-1α protein. HaCaT cells were treated with Cup, AirP, and PC for 5 minutes. After 2 hours, the location of HIF-1α (green) was monitored using confocal microscopy as described in the Materials and Methods section. DAPI was used to counter stain the nuclei of cells. **(B)** HaCaT cells were transfected with scrambled RNA or siRNA against HIF-1α as described. At 24 hours post-transfection, the cells were treated or non-treated with PC for 5 minutes. After 4 or 6 hours of further incubation, the cells were harvested, and total protein and RNA were used for western blot (top 2 panels) or RT-PCR (bottom 2 panels) analysis, respectively. Data shown are the representatives of three independent experiments.

To test whether the PC-mediated activation of ERK is directly linked to the HIF-1α protein activity, the effects of cupping, airP, and PC on HIF-1α protein localization was monitored (Fig. 4A). As our data shows, HIF-1α protein was rarely visible in non-treated HaCaT cells. The cupping treatment did not affect HIF-1α protein. However, treatment with airP slightly increased the number of nuclear HIF-1α harboring cells, and the majority of HaCaT cells had nuclear HIF-1α after PC treatment.

In other to confirm whether HIF-1 is essential for the PC-mediated increase of VEGF-A, siRNA against HIF-1α was utilized. As Fig. 4B shows, PC treatment of HaCaT cells transfected with scrambled RNA induced VEGF-A mRNA along with HIF-1α protein, but the use of siRNA against HIF-1α completely blocked the PC-mediated induction of VEGF-A.

### Elevation of NO in PC-treated media might be responsible for PC-mediated VEGF-A expression

**Figure 5:**
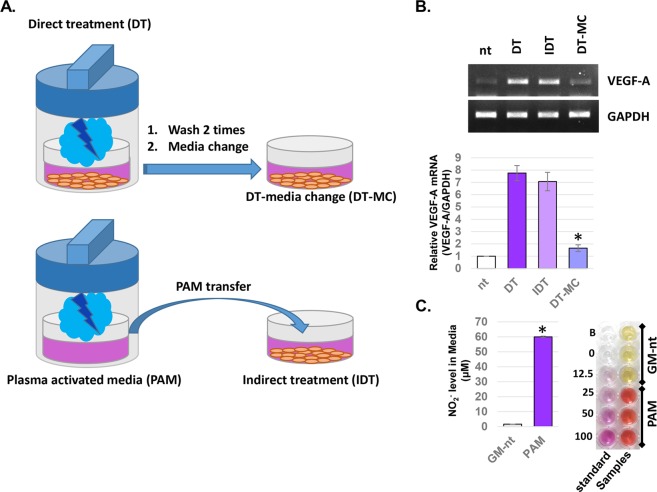
PC-mediated increase of NO within the media might be responsible for the PC-mediated VEGF-A gene expression. (**A)** A schematic figure describing three different cell treatment methods with PC. **(B)** At 6 hours post-treatments of cells by three different PC treatment methods, total RNA was extracted and used for RT-PCR analysis. Data shown are the representatives of three independent experiments, *p < 0.005. DT: direct treatment; IDT: indirect treatment; DT-MC: direct treatment and media change. **(C)** The concentration of NO_2_^−^ level in the cell growth media was detected. Cell growth media (2 ml) was treated with PC for 5 minutes to make plasma-activated media (PAM) and then immediately used for a Griess assay. Data shown are the average of three independent experiments, *p < 0.0005.

To elucidate the working elements of PC in VEGF-A regulation, three different types of PC treatment methods were adopted as described in Fig. 5A. In all previous experiments, the cells were treated with PC in the presence of 2 ml of growth media within the cup, and this method was identified as ‘direct treatment’ (DT). The cells treated with DT would be exposed to all of the working elements of PC along with the vacuum required for cupping. To remove the direct effects of the physical working elements of PC such as UV, electric fields, and a vacuum, the cell growth media was separately treated with PC for 5 minutes to create plasma-activated media (PAM). Then, the cells were treated with PAM, and this treatment method was identified as ‘indirect treatment’ (IDT). Conversely, the last treatment method, DT and media change (DT-MC) can minimize the effects of chemical working elements that can be melted into or modulated by PC such as reactive species and ions. At 6 hours after the three different PC treatments, the expression level of VEGF-A gene was analyzed (Fig. 5B). Interestingly, IDT treatment of cells with PC showed an induced level of VEGF-A mRNA similar to that of DT-treated cells (about 7.1- and 7.8-fold induction on average respectively, but there were no significant differences). Conversely, the cells treated with DT-MC methods showed only a slight increase (about 1.66 fold) in VEGF-A gene expression.

To test if the elevated levels of excited N_2_ generation from PC can modulate RNS levels within the cell growth media, the media was treated or non-treated with PC for 5 minutes, and the level of nitrite ion (NO_2_^−^) was measured. As shown in Fig. 5C, the NO_2_^−^ level in normal growth media was about 3 μM, but it was elevated to about 60 μM by PC treatment. To evaluate the effect of the components in the cell growth media on the PC-mediated NO_2_^−^ production, cell growth media (GM) with 10% FBS, serum free media (SFM) and DPBS was subjected to 5 minutes of PC treatment and then the NO_2_^−^ levels were monitored. As our Supplementary Data 1 shows, the NO_2_^−^ level of PC treated SFM was similar (92.7%) to that of PC treated GM, but the NO_2_^−^ level in PC treated PBS was about a half (51.2%) to that of PC-treated GM. These data not only prove the fact that the elevated NO_2_− in the PC-treated GM is not an artifacts of Griess assay which can be caused by proteins in the serum, a method used for detecting NO_2_−, but also suggest that the non-protein components of GM are the source for half of PC-mediated NO_2_− generation.

## Discussion

During the 2016 Olympic season in Brazil, the American team stated that the use of cupping was an efficient way to reduce muscle pain caused by hard training. It is well known that even Michael Phelps, a highly decorated swimmer, is a proponent of cupping. Actually, the effectiveness of cupping is controversial. Some say it is helpful while others remain skeptical. Although cupping therapy has been used for thousands of years, its effectiveness is still under debate. The specific working mechanism of cupping has not yet been scientifically explained, which is why some scientists regard cupping therapy as pseudoscience.

In this study, we examined the beneficial effects of a new type of cupping called PC, which is traditional cupping combined with non-thermal plasma. As our data shows, the plasma-generating efficacy in a cupping-like semi-vacuum condition is much higher than that of atmospheric pressure air plasma under the same input power condition (Fig. 1B). Furthermore, several excited secondary N_2_ systems were generated more efficiently by PC than atmospheric pressure air plasma. This means the medicinal effects of the plasma can be increased under the cupping condition too. Since the nitrogen species are well known for their role in the wound healing process^14^, several wound healing related cytokines and growth factors might be affected by PC. Among them, we focused on VEGF, since it might accelerate the healing of damaged blood vessels after traditional cupping therapy. In our results, the mere treatment of human keratinocytes with cupping rarely affected VEGF-A expression. This result is in line with the result of Wang *et al*., who showed slight increase (about 1.3 fold) of VEGF-A in pig wound tissue under the vacuum condition using a vacuum-assisted closure (VAC) device^15^. Conversely, the treatment of cells with airP significantly increased the expression of VEGF-A, and the effectiveness of plasma-mediated regulation of VEGF-A became enhanced under the cupping condition. The treatment of PC on HS68 human dermal fibroblast also increased the expression of VEGF-A (Supplementary Data 2). These results demonstrates that the most well-known property of cupping, blood perfusion in the lesion, can be significantly enhanced by VEGF-mediated angiogenesis when plasma was added. In our previous report, the effect of non-thermal argon plasma under the atmospheric pressure condition significantly induced VEGF-A expression from skin cells^13^. However, the effects of plasma on the expression of other genes related to angiogenesis were not explored, and the specific mechanism of plasma-mediated VEGF-A regulation was not elucidated. Interestingly, PC treatment only induced the expression of VEGF-A gene among other VEGF family members and FGF-2, another angiogenesis-promoting growth factor (Supplementary Data 3).

Since VEGF-A is well known for its enhanced expression after activation of the transcription factor HIF-1, the effect of a vacuum and PC on HIF-1α protein expression was tested (Fig. 2). HIF-1 plays an important role during the wound healing process; as a transcription factor, it stimulates several kinds of wound healing promoting genes including VEGF-A, collagen, and nitric oxide synthase^16^. The activity of HIF-1 is primarily governed by the status of its α subunit, because the protein stability of HIF-1α is dependent on oxygen levels^17^. Under normal conditions, HIF prolyl-4-hydroxylases (PHDs) rapidly hydroxylates HIF-1α, and then von Hippel-Lindau (VHL) protein promotes ubiquitin-mediated protein degradation of HIF-1α. However, under hypoxic conditions or during wound healing, HIF-1α becomes stabilized as PHD enzyme are not functioning. The result of this study showed that isolated treatment of cupping on keratinocytes did not induce the expression of HIF-1α. Conversely, the treatment of cells with airP and PC increased HIF-1α levels by 1.7 fold and 2.6 fold, respectively. However, the PC-mediated increase of HIF-1α seems to be mediated by a minor HIF-1α regulating program since the induction rate of HIF-1α after PC treatment was extremely low in comparison to the effect of cobalt chloride, an iron chelator that can inhibit PHD activity^18^. Interestingly, along with a slight increase of HIF-1α after PC treatment, the activities of JNK and ERK were also increased. The data show that the activation of JNK and ERK could be responsible for the PC-mediated increase of HIF-1α and VEGF-A expression.

Interestingly, our results showed that rather than the increase in HIF-1α levels, the activity of ERK was crucial for PC-mediated VEGF-A expression. The ERK inhibitor, not the JNK inhibitor, completely blocked the PC-mediated induction of VEGF-A, although the induction of HIF-1α after PC treatment still occurred (Fig. 3A). This result is in line with the report of Minet *et al*., who elucidated the critical role of ERK activation for HIF-1 activation under hypoxic conditions^19^. Therefore, the treatment of the cells with PC might activate HIF-1α through the activation of ERK, which was directly linked to the increased VEGF-A expression. This possibility was further confirmed by our result in Fig. 4. The treatment of cells with PC induced the nuclear-localization of HIF-1α, and the PC-mediated increase of VEGF-A gene expression was completely blocked by the use of siRNA against HIF-1α.

In the last part of this study, the possible key working element of PC for VEGF-A gene expression regulation was suggested. As a type of negative pressure plasma, PC ejects several working elements which can be divided into physical (UVs, electric fields) and chemical elements (charged particles, reactive nitrogen and oxygen species). Among these various working elements, UVs are well known for inducing VEGF-A expression in several types of cells, including keratinocytes and fibroblasts^20–23^. However, in Fig. 5A,B, we determined that the treatment of cells with PC-treated media had similar VEGF-A inducing activity compared to direct treatment of cells with PC. Furthermore, the immediate removal of PC-treated media after the direct treatment significantly blocked the PC-mediated increase of VEGF-A. These data represent the fact that the physical elements, including UVs generated from PC, are not the key elements for the PC-mediated VEGF-A gene expression. To investigate how PC could activate ERK activity, we examined nitric oxide (NO), the most prominent candidate for this, and it can be melted into the cell growth medium after PC treatment. As described in our results of OES analysis, the main active species of PC was the secondary N_2_ system, not reactive oxygen species (Fig. 1C). This elevated production of excited N_2_ might cause the increase of NO within the media (Fig. 5C), and this might be directly linked to the ERK-mediated HIF-1α activation, which is directly linked to VEGF-A expression (Supplementary Data 1B). Many other researchers have reported that the relationship between NO and HIF-1-mediated VEGF-A expression. Kimura *et al*. reported that treatment of cells with NO donor, SNAP, and NOC5 increased VEGF-A promoter activity by stimulating the DNA binding of HIF-1 protein^24^. In addition, Palmer *et al*. reported that among several kinds of NO donors, the donors that can release electrophilic NO, NO^+^, are more potent than the neutrophilic NO donors for HIF-1 activation^25^. Notably, Kasuno *et al*. reported that NO-donor-mediated activation of HIF-1α is dependent on MAPK/ERK signaling^26^. The results of this study suggest that reactive nitrogen species (RNS) generated from PC might stimulate the generation of NO within the media, which might be responsible for the PC-mediated ERK activation and HIF-1-mediated VEGF-expression. Interestingly, in this study the effect of PC on ERK-HIF-1α and VEGF-A was significantly reduced under the presence of NAC, a pan-ROS scavenger (Fig. 3B). It is well known that the treatment of NO donor to various types of cells can increase cellular ROS, and this was directly linked to the biological function of NO^27,28^. In addition, Liu *et al*. found that the activation of ERK and HIF-1α under hypoxic conditions can be blocked by NAC, and the isolated treatment of H_2_O_2_ can efficiently activate ERK and HIF-1α^29^. Similar to these reports, PC treatment also increased the cellular ROS level although PC did not produce OH radicals (Supplementary Data 4). Therefore, the fact that the effect of PC can be reversed by the use of NAC suggests that the PC treatment might first increase the NO level, which in turn stimulates the accumulation of cellular ROS that can be directly linked to ERK and HIF-1α activation.

Until now, traditional cupping has failed to persuade skeptics, as the scientific evidence supporting a mechanism of action was not sufficient. Here, we merged traditional cupping with the new medical technology, NTP. This study was primarily focused on adding angiogenesis-promoting properties of NTP to traditional cupping. Furthermore, the specific molecular mechanism for PC-mediated expression of VEGF-A was investigated. As PC stimulated activation of HIF-1, which is very important for enhancing wound healing activity, PC was expected to accelerate the healing duration after skin tissue damage mediated by cupping. In our previous study, we reported the strong anti-inflammatory activity of NTP^12^. As our Supplementary Data 5 shows, PC also effectively blocked TNFα-mediated pro-inflammatory cytokine expression in HaCaT cells. This result indicates that PC not only stimulates angiogenesis and wound healing but may also be helpful for treating inflammatory muscle pain. Taken together, this study represents scientific evidence for the medicinal action of PC, the advanced form of traditional cupping.

## Methods

### Reagents

All chemicals were purchased from Sigma-Aldrich, Korea unless otherwise indicated.

### Cell culture

HaCaT human keratinocytes were maintained in Dulbecco’s Modified Eagle’s Medium (DMEM, Gibco, Grand Island, NY, USA) supplemented with 1% penicillin/streptomycin (Gibco) and 10% fetal bovine serum (FBS, Gibco). Cells were incubated at 37 °C and 5% CO_2_.

### PC device

The new type of plasma cupping device system (Fig. 1A) was developed for this study with the support of ICD incorporation (Ahn-Seong, Kyeong-Gi-do, South Korea). A circular-type plasma source inside the plasma cupping device was a surface dielectric barrier discharge (DBD) type, which consisted of two electrodes and one dielectric layer. The circular dielectric plate was made of TLA-5A (Taconic Inc., Petersburgh, NY, USA) material, and its thickness is 0.254 mm to fit the relative dielectric constant at 2.2. The electrodes were covered on both sides of a dielectric plate that was made of copper plated with gold at the thickness of 2 µm. The sinusoidal high voltage, up to 10 kV_pp_, with a frequency of 20 kHz was applied to the top electrode, while the bottom electrode was grounded to prevent an electric shock between the plasma source and human skin.

### OES analysis

Optical emission spectroscopy (OES) was observed using a spectrometer (USB2000+, Ocean Optics, Largo, FL, USA) in the wavelength range of 200–800 nm. The emission spectra of the plasma source observed at a pressure of 760 Torr and 410 Torr are shown in Fig. 1C. The spectra of N_2_ emission bands with a wavelength of 310–440 nm and N_2_^+^ emission bands with a wavelength of 391–428 nm were observed prominently from both cases.

### PC treatment of cells

HaCaT cells were seeded at 5 × 10^5^ in a 35 mm cell culture dish and incubated for 48 hours. Immediately prior to the treatment, the growth media was changed with 2 ml of fresh growth media and placed on a thick rubber plate in order to form the appropriate strength of vacuum within the cup. After the cup was placed on a dish, the PC treatment process was assessed for 5 minutes. After the treatment, the cells were incubated in the CO_2_ culture chamber for the indicated times.

### RT-PCR

At 6 hours after the treatment, the cells were washed with ice-cold phosphate-buffered saline (PBS), and total RNA was extracted using TRIzol^TM^ reagent (Thermo Fisher Scientific) as described in the manufacturer’s manual. The specific methods for cDNA synthesis and PCR can be found in our previous report. In brief, cDNA synthesis was assessed using a Maxime RT PreMix kit (iNtRON Biotechnology, Seoul, South Korea), and this cDNA was used for PCR against GAPDH and VEGF-A.

### Western blot analysis

After a final incubation, the cells were washed with PBS and harvested in ice-cold lysis buffer containing 50 mM Tris/HCl (pH 7.5), 150 mM NaCl, 1% (v/v) Nonidet P40, 10% (v/v) glycerol, 1 mM PMSF, 1 mM dithiothreitol, 20 mM NaF, 1 mM EDTA, and a protease inhibitor cocktail (Roche). Equal amounts of cell lysate (30 μg) were resolved by SDS/PAGE (8–12% gel) and transferred to PVDF membranes. Upon the completion of transfer, the membranes were probed with antibodies against extracellular signal-regulated kinase (ERK), phospho-ERK, c-Jun N-terminal kinase (JNK), phospho-JNK (Santa Cruz Biotechnology, Santa Cruz, CA, USA), and hypoxia inducible factor 1 α (HIF-1α, BD Bioscience, Franklin Lakes, NJ, USA). The specific bands were detected with advanced ECL western blotting reagents (Merck Millipore, Darmstadt, Germany). An anti-GAPDH antibody was used as a loading control (Santa Cruz Biotechnology).

### Immunofluorescence assay

Two days before the treatments, 5 × 10^4^ of HaCaT cells were seeded in a glass-bottom cell culture dish for confocal microscopic observation. Two hours after the indicated treatments, the cells were fixed using 4% paraformaldehyde. Then, the cells were permeabilized with 0.5% Tween 20 and blocked with 10% BSA for 1 hour at room temperature. Thereafter, the cells were incubated with a monoclonal antibody against HIF-1α at 4 °C overnight. After washes with PBS three times, the cells were incubated with Alexa 594-conjugated goat anti-mouse IgG antibody (Invitrogen, Carlsbad, CA, USA, 1:200) for 1 hour at room temperature. The expression of E-cadherin was detected under the confocal microscope. DAPI was used to counterstain the nuclei.

### siRNA experiments

A day before the transfection, 2 × 10^5^ of HaCaT cells were seeded in a 35 mm dish. The transfection of cells with siRNA against the HIF-1α gene was performed using a siRNA transfection reagent kit (Santa Cruz Biotechnology) according to the manufacturer’s manual. A day after the transfection, the cells were treated or non-treated with PC for 5 minutes, and the cells were harvested at 4 hours (for western blot) and 6 hours (for RT-PCR) after the PC treatment.

### Griess assay

To evaluate the effect of PC on the NO density within the cell growth media, the Griess assay was adopted using a Griess Reagent Kit (Invitrogen, Eugene, OR, USA) according to the manufacturer’s protocol. In brief, 2 ml of cell growth media was added to a 35 mm cell culture dish and treated with PC for 5 minutes. Immediately after the treatment, 150 μl of non-treated and PC treated media was used for the Griess assay (in triplicate). After 30 minutes of incubation, the color changes were photographed and the optical density of the media was read by 96-well microplate reader at 548 nm.

### Data analysis

Data are presented as the mean ± standard error of the mean (SEM) of at least three independent experiments. The two-tailed Student’s *t*-test was used to assess statistical significance for differences in mean values, and the significance was set at p < 0.05.

## Supplementary information

Supplementary Data
