# Supplementary material for: Plasma cupping induces VEGF expression in skin cells through nitric oxide-mediated activation of hypoxia inducible factor 1
Source: Sci Rep. 2019 Mar 7;9:3821. doi: 10.1038/s41598-019-40086-8 (PMC6405951; doi:10.1038/s41598-019-40086-8)

Plasma cupping induces VEGF expression in skin cells through nitric oxide-mediated activation of hypoxia inducible factor 1

Hyun-Young Lee[1], Hae-June Lee[1], Gyoo-Cheon Kim[2,4], Jeong-Hae Choi[2,3*] and Jin-Woo Hong[3*]

[1] Department of electrical engineering, Pusan National University
[2] Department of Anatomy and Cell Biology, School of Dentistry, Pusan National University
3 Department of Internal Medicine, School of Korean Medicine, Pusan National University

* These authors are contributed equally for this study.

Corresponding authors:

Jin-Woo Hong, Department of Internal Medicine, School of Korean Medicine, Yangsan Campus of Pusan National University, Beomeo-ri, Mulgeum-eup, Yangsan-si, Gyeongsangnam 626-870, South Korea, e-mail: jwhong@pusan.ac.kr

Jeong-Hae Choi, Department of Anatomy and Cell Biology, School of Dentistry, Yangsan Campus of Pusan National University, Beomeo-ri, Mulgeum-eup, Yangsan-si, Gyeongsangnam 626-870, South Korea, e-mail: monday27@pusan.ac.kr

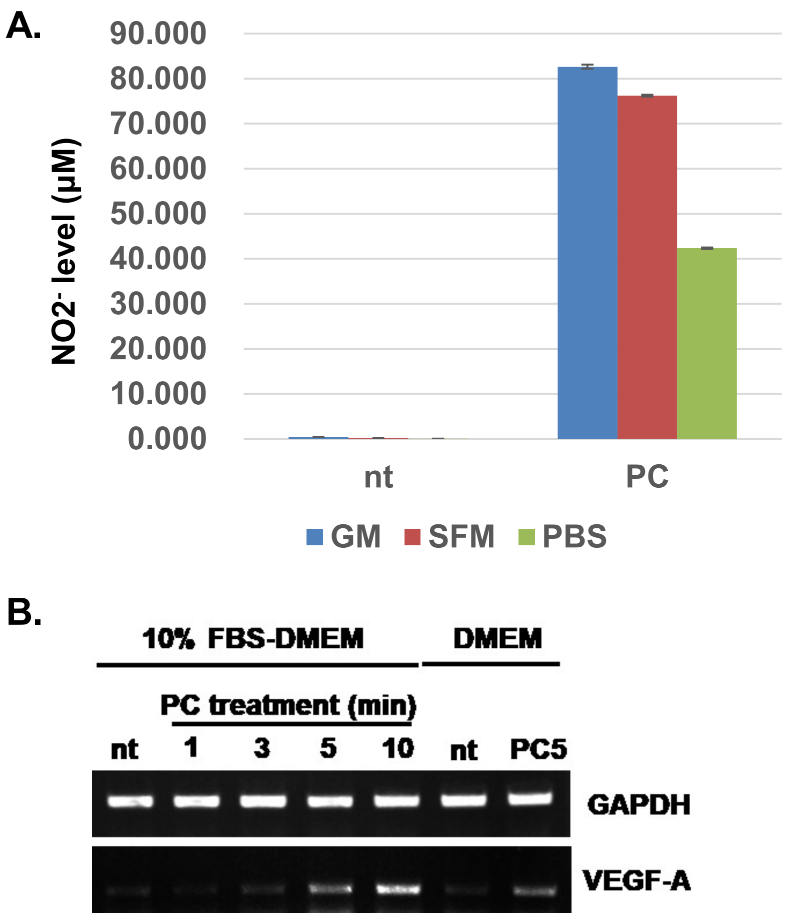

Supplementary Data 1. PC treatment induces VEGF-A expression in serum-independent manner by increasing NO_2^- level in the media. (A) Cell growth media (GM: 10% FBS containing DMEM), serum fre media (SFM) and DPBS were subjected to PC treatment for 5 minutes, and then NO_2^- level was detected using Griess assay. (B) HaCaT cells were subjected to PC treatment for indicated times in the presence ore absence of 10% FBS, and the expression of VEGF-A gene was monitored at 6 hours after the treatment. The data shown are the representatives of 3-independent experiments.

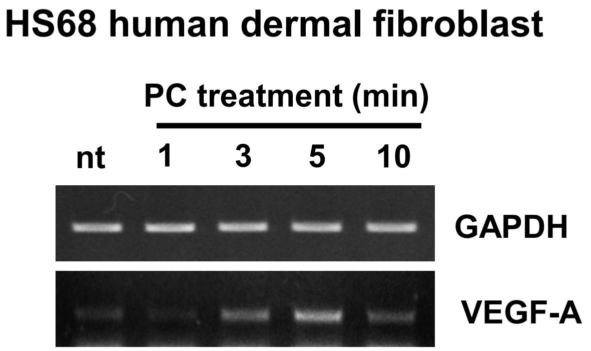

Supplementary Data 2. PC treatment on HS68 human dermal fibroblasts induced the VEGF-A gene expressions. HS68 cells were subjected to PC treatment for indicated times, and the expression of VEGF-A gene was monitored at 6 hours after the treatment. The data shown are the representatives of 3-independent experiments.

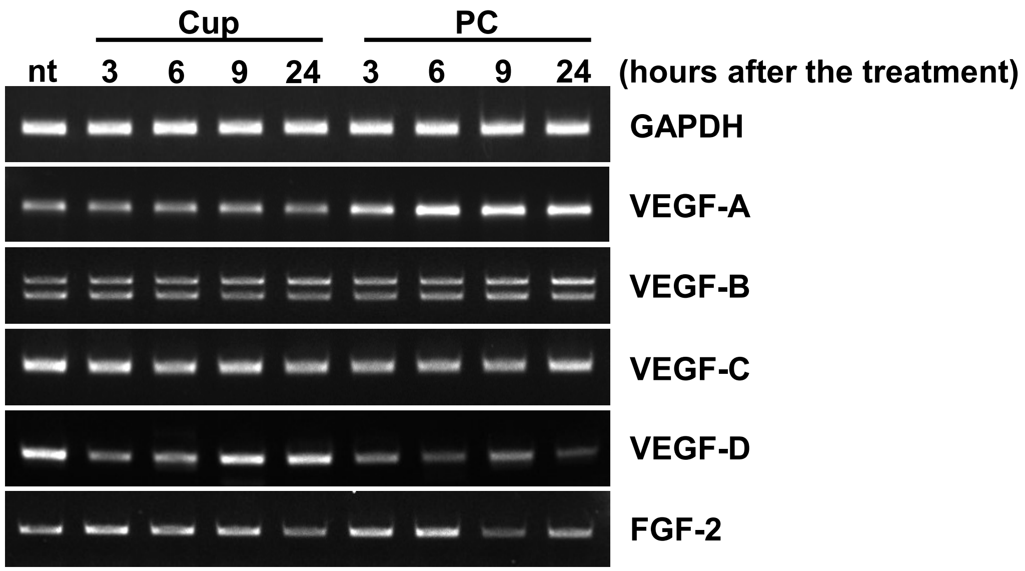

Supplementary Data 3. VEGF-A gene is a major target of PC among the angiogenesis-related genes. HaCaT cells were subjected to 5 minutes of cupping and PC treatment, and the expression pattern of angiogenesis-related genes were monitored at 3, 6, 9 and 24 hours after the treatment. The data shown are the representatives of 3-independent experiments.

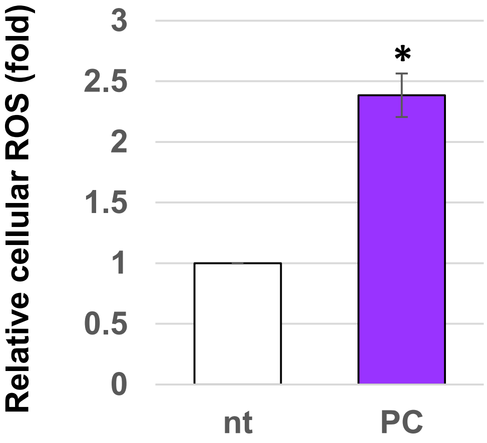

Supplementary Data 4. PC treatment increases cellular ROS level. HaCaT cells were incubated in PC-treated (5 minutes) media for 4 hours, and then the cellular ROS level was measured using H2DCFDA (Invitrogen) as manufacturer's manual. Data shown are representatives of three independent experiments, * $p<0.05$.

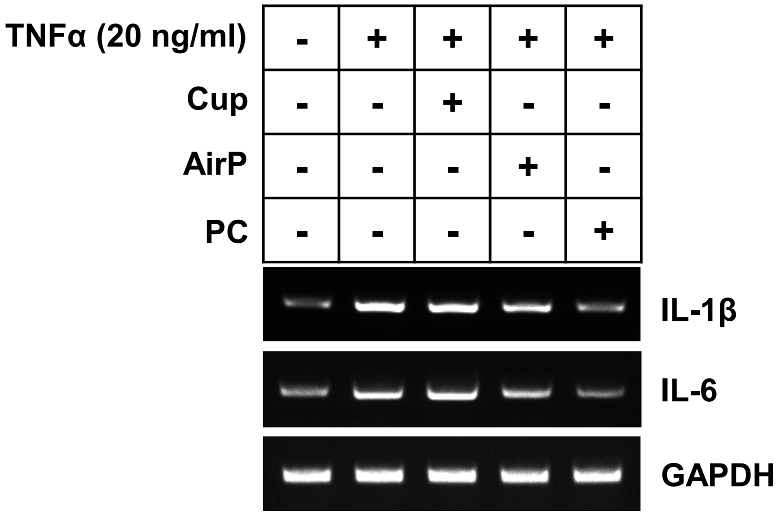

Supplementary Data 5. PC treatment can block TNFα-mediated expression of pro-inflammatory cytokines. HaCaT cells were incubated under presence or absence of TNFα (20 ng/ml) for 18 hours and then subjected to 5 minutes of cupping, airP and PC treatment as indicated. The expression pattern of IL-1β and IL-6 was monitored at 6 hours after the treatment. The data shown are the representatives of 3-independent experiments.

Supplementary information: Full gel Images

Figure 1D

GAPDH (289 bp)

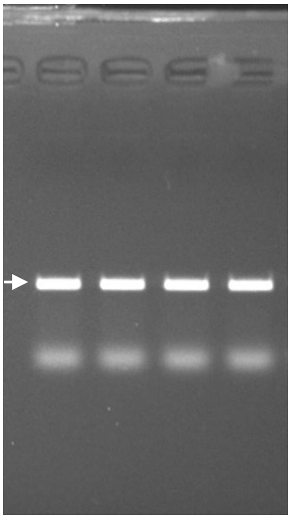

VEGF-A (307 bp)

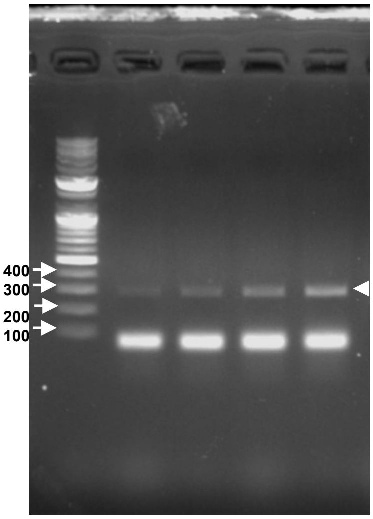

Supplementary information: Full gel Images

Figure 2A

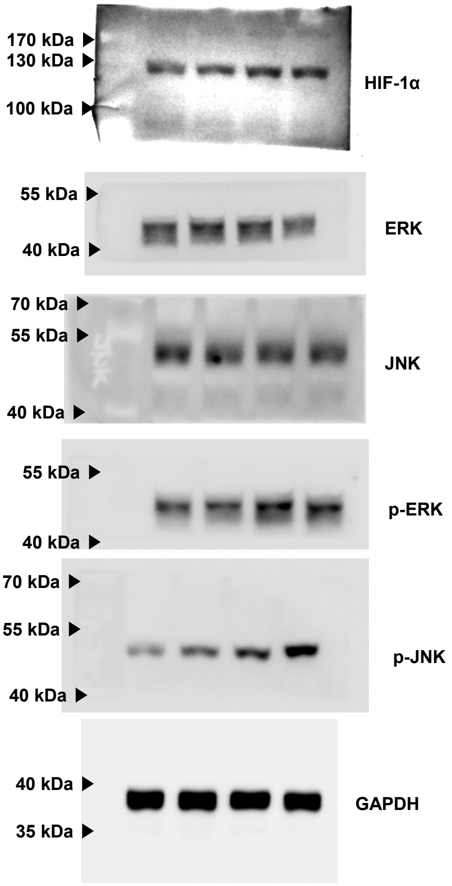

Figure 2C

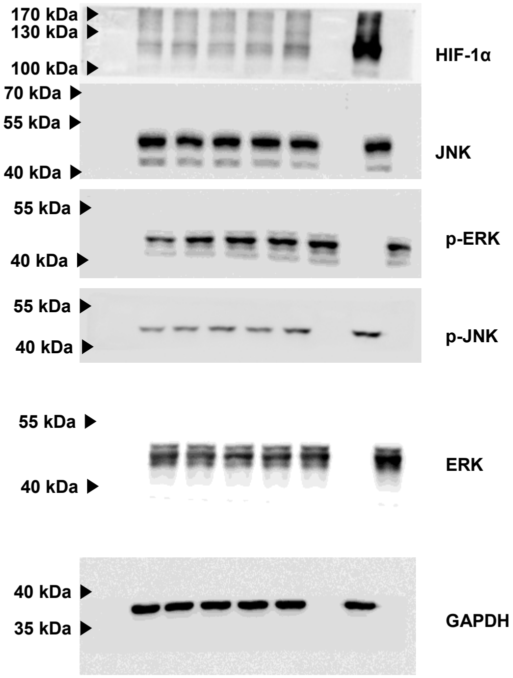

Supplementary information: Full gel Images

Figure 3A

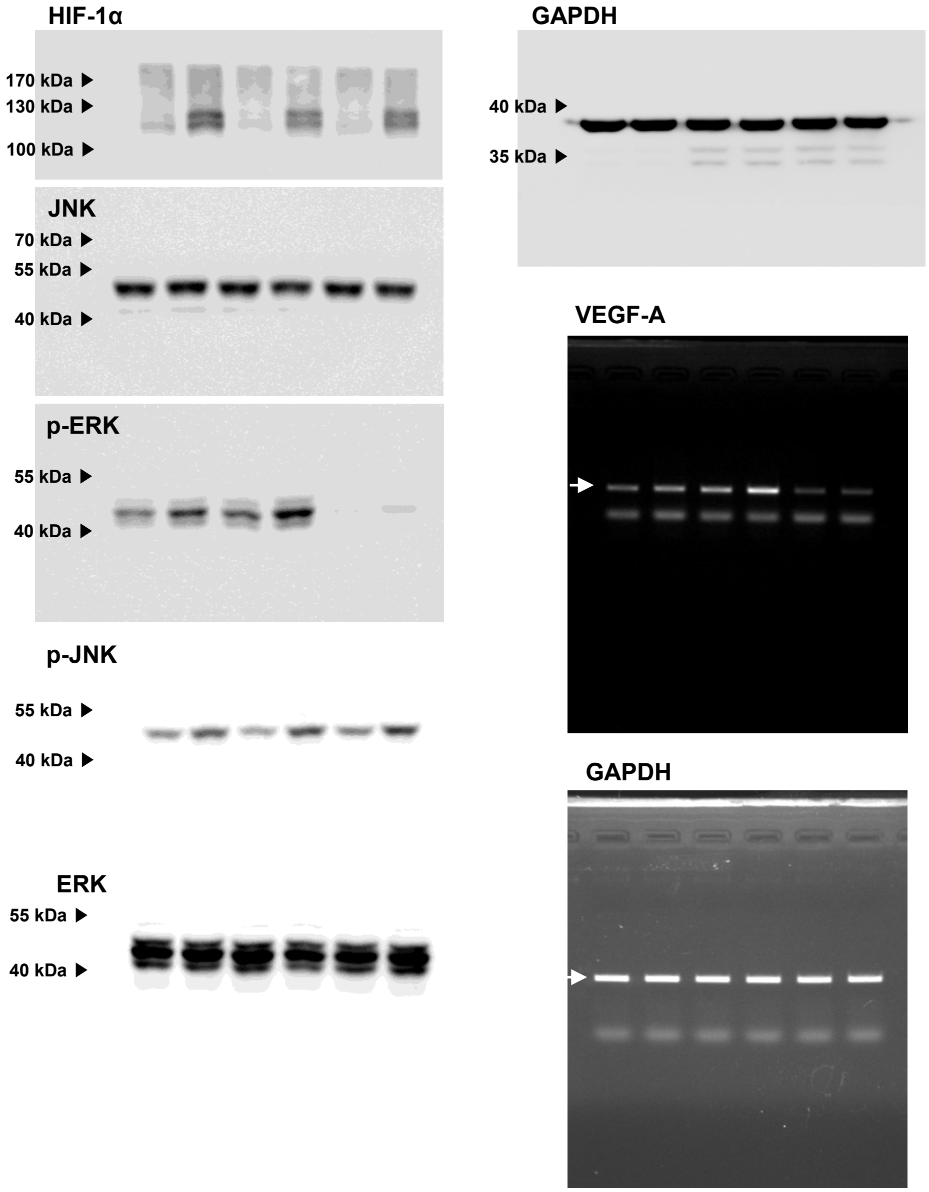

Supplementary information: Full gel Images

Figure 3B

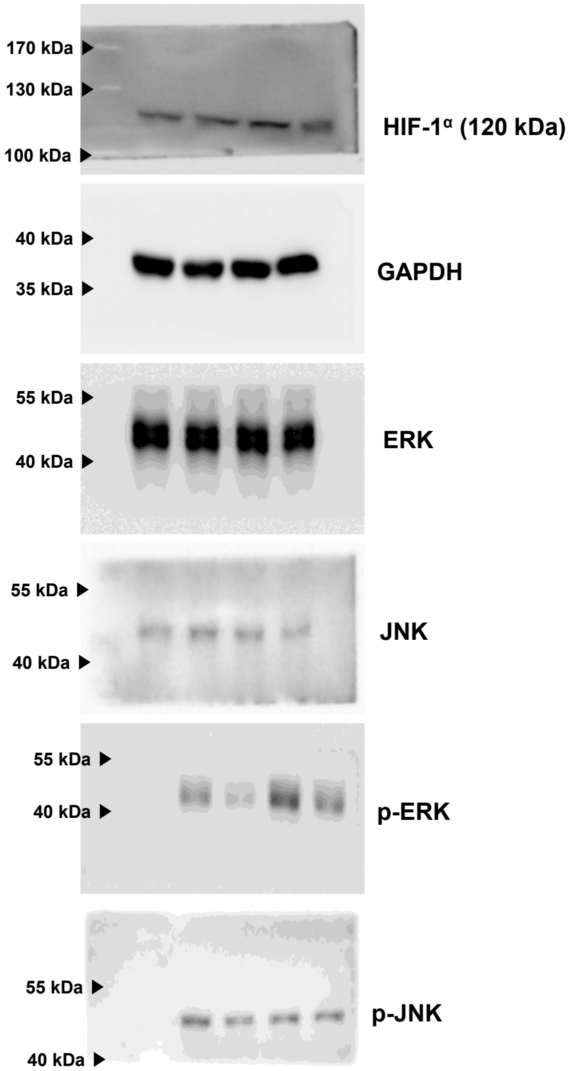

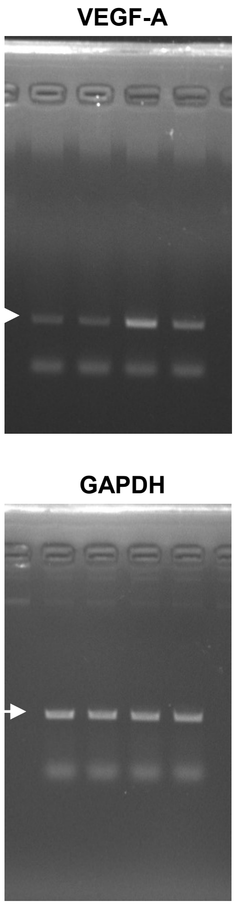

Supplementary information: Full gel Images

Figure 4B

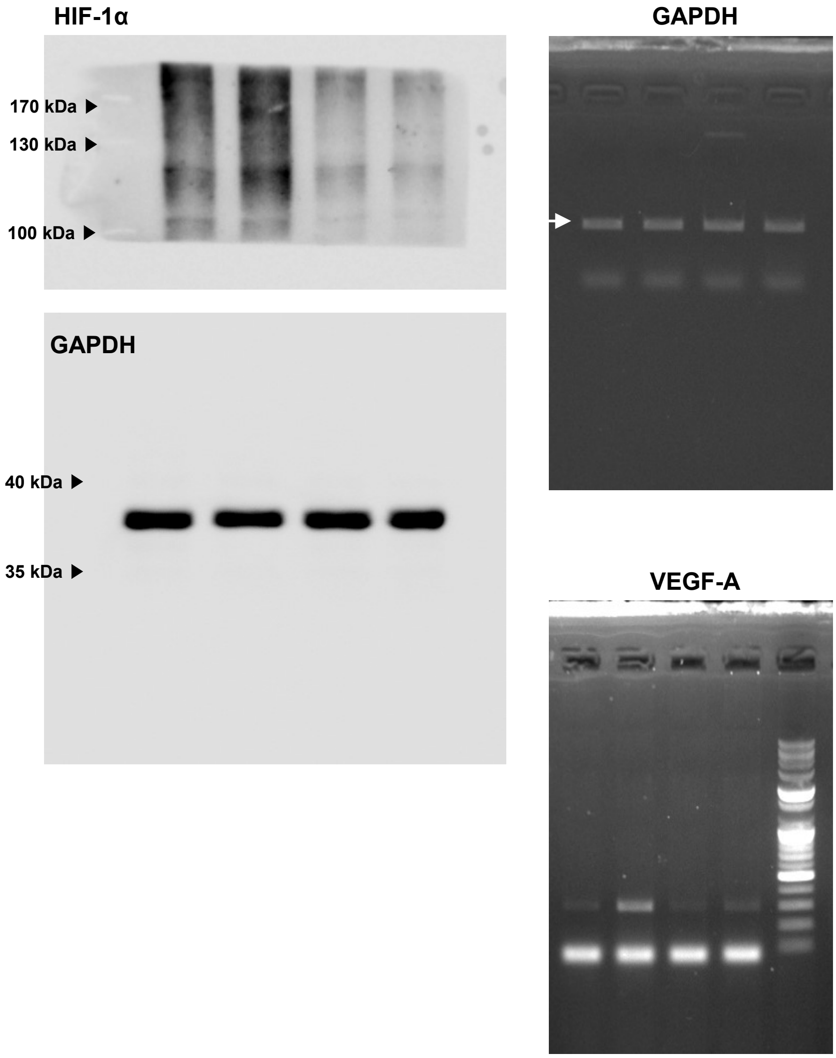

Supplementary information: Full gel Images

Figure 5B

VEGF-A

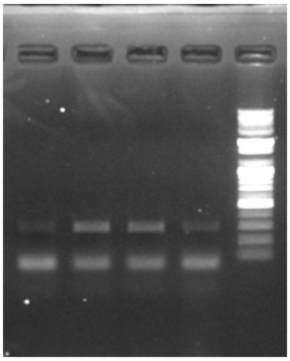

GAPDH

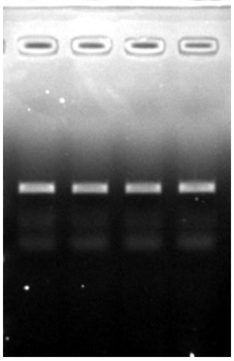

Supplement: Supplementary file 1 — Supplementary Data [file 41598_2019_40086_MOESM1_ESM.pdf]
